# A prospective histopathological analysis of the inferior turbinate: which functional parts should be preserved during turbinate surgery?

**DOI:** 10.1016/j.bjorl.2024.101486

**Published:** 2024-08-09

**Authors:** Salma Saud AlSharhan, Norah Mohammad AlNafea, Mona Mohammad Ashoor, Wasan Fahad AlMarzouq, Salwa ALRashed AlHumaid, Nada Abdallah ALBahrani, Areej Manssour Al Nemer, Reem S. AlOmar, Hussain Jawad Aljubran

**Affiliations:** a Imam Abdulrahman Bin Faisal University, College of Medicine, Department of Otorhinolaryngology, Dammam, Saudi Arabia; b King Faisal Specialist Hospital and Research Center, Department of Otorhinolaryngology, Riyadh, Saudi Arabia; c King Abdulaziz Medical City, National Guard Health Affairs, Department of Otorhinolaryngology, Jeddah, Saudi Arabia; d Imam Abdulrahman Bin Faisal University, College of Medicine, Department of Pathology, Dammam, Saudi Arabia; e Imam Abdulrahman Bin Faisal University, College of Medicine, Department of Family and Community Medicine, Dammam, Saudi Arabia

**Keywords:** Histopathology, Hypertrophy, Inferior turbinate, Surgical technique, Turbinoplasty

## Abstract

**Objective:**

Inferior turbinate (IT) hypertrophy-induced chronic nasal obstruction is one of the most common problems in rhinology. However, the histopathological analysis of the hypertrophic IT is unclear. Therefore, this study aimed to identify the histological changes and the most functional areas of the IT to assist otolaryngologists with improving and modifying surgical techniques and minimizing potential complications.

**Methods:**

This prospective, cross-sectional study was conducted to evaluate the contribution of hypertrophic IT to nasal obstruction. For the analysis, a total of 38 adult patients (IT hypertrophy group and non-IT hypertrophy [control] group) were enrolled, and 131 specimens were obtained during the surgical procedures (IT hypertrophy group, endoscopic submucosal turbinoplasty and septoplasty; non-IT hypertrophy group, septoplasty). Intraoperative samples were collected from four sites of the IT to determine the dimensions, composition, and possible pathological changes in each individual site. The samples were analyzed using light microscopy.

**Results:**

A comparison of the four sites of the IT in the IT hypertrophy group showed that the posterior end had the highest normal epithelium percentage, and cilia count. This suggests that preserving the functional part of the IT during surgery is crucial. Furthermore, a comparison of both groups in terms of basement membrane thickness and vessel wall thickness (*p* = 0.005 and *p* = 0.03, respectively) showed significant differences.

**Conclusion:**

Our findings can assist otolaryngologists select the most appropriate surgical procedures for IT hypertrophy. In addition, they advocate the importance of preserving the functional part of the IT during surgical intervention to achieve an efficiently working IT and avoid undesirable complications while improving the nasal airway passage.

**Level of evidence:**

Level 3.

## Highlights

• Histopathological analysis of IT hypertrophy remains insufficient.
• Patients with IT hypertrophy had higher basement membrane thickness.
• The posterior end of IT had the highest normal epithelium percentage.

## Introduction

Chronic nasal obstruction caused by inferior turbinate (IT) hypertrophy is one of the most common problems encountered in rhinology.^1^ However, there is no consensus regarding the optimal removal of turbinate tissue during surgical interventions, and there exists the possibility of recurrence.^2^

Many nasal mucosal diseases, such as allergic rhinitis and chronic rhinosinusitis, are directly related to structural mucosal changes of the nasal turbinate. Morphological and histological studies of the nasal turbinate have enriched our knowledge of the physiology of the nose and helped with the development of different therapeutic measures for treating diseases of the nasal mucosa.^3^

Numerous surgical procedures for the IT with the aim of reducing the turbinate size and improving nasal patency have been proposed in the literature. These include cauterization, coblation, radiofrequency reduction, microdebrider resection, and partial or total turbinate resection.^4^ However, excessive resection of the nasal mucosa may cause nasal dryness and crusting, which can alter the airflow. Therefore, one of the main goals of surgical treatment for nasal mucosal diseases is to preserve the nasal mucosa to achieve a balance between improving the nasal obstruction caused by allergy or rhinitis and avoiding dryness resulting from excessive surgical removal of the mucosal lining (atrophic rhinitis).^3^

The submucosal turbinoplasty technique has been proposed for the management of IT hypertrophy in allergic rhinopathy patients because it has been advocated that this technique preserves the respiratory epithelium and decreases the number of inflammatory cells in the nasal mucosal lining.^5^ However, others prefer to study the physiology and histopathology of the nasal mucosa before planning the surgical technique.^6^

To the best of our knowledge, the available studies lack both quantitative and qualitative information regarding the composition and histological features of the nasal mucosa at different sites of the nasal cavity. Moreover, there are insufficient data regarding whether these changes are permanent or reversible. Hence, this morphometric study is among the first to evaluate the dimensions and composition of the IT and analyze the histopathology of different sites of the enlarged IT. These data will assist surgeons with determining and conserving the most functional sites of the IT and with performing effective surgical turbinoplasty that preserves the IT.

## Methods

Ethical approval was obtained from the institutional review board (IRB) of Imam Abdulrahamn bin FaisaL University, and informed consent was obtained from all patients prior to their enrollment (IRB-2018-01-240). This study did not interfere with the patients’ previously proposed management or affect their care.

### Study population

38 patients from the Department of Otorhinolaryngology at 38 patients from the Department of Otorhinolaryngology at King Fahd University Hospital were enrolled between January and July 2019. The inclusion criteria were nasal obstruction, age between 18 and 65 years, and nasal endoscopic examination findings of IT hypertrophy (score 3; posterior wall of the nasopharynx not visible). However, the posterior wall of the nasopharynx could be seen through ≥50% of the posterior choana (score 1) in the control patients.^7^ All patients who had undergone previous nasal surgery, had underlying disorders (e.g., Wegener’s granulomatosis, sarcoidosis, cystic fibrosis, and Kartagener’s syndrome), had a Lund-MacKay computed tomography (CT) score more than 2 on both sides, or had missed data were excluded. Accordingly, 4 patients IT hypertrophy group were excluded.

We hypothesized that most of the patients (IT hypertrophy group) had similar conditions of persistent IT hypertrophy and nasal obstruction for more than 3 months that did not respond to medical treatment such as intranasal topical steroids or oral antihistamines, resulting in their presentation to a tertiary hospital.

### Specimen collection and tissue preparation

Data were collected from the included patients during their proposed nasal surgeries to determine the dimensions, composition, and possible histopathological changes of different sites of the IT.

**Figure 1:**
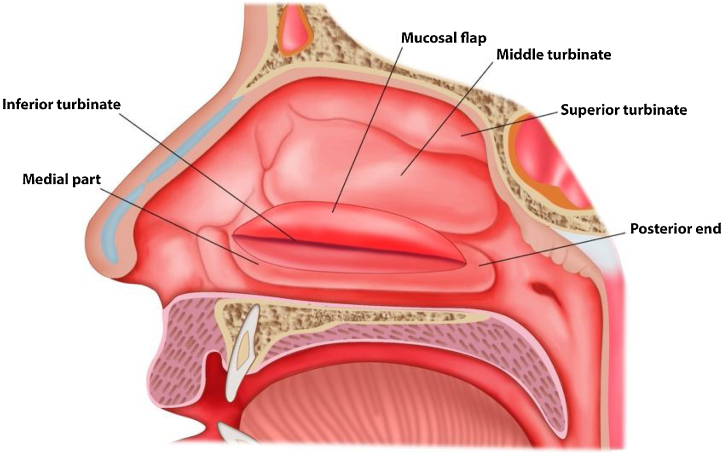
Illustration of the surgical approach of turbinoplasty which was performed in the study.

The surgical procedures included endoscopic submucosal turbinoplasty with or without septoplasty for the IT hypertrophy group. Turbinate tissue specimens were obtained during septoplasty of the control group patients. The analyzed material comprised a total of 131 samples. We were able to obtain four specimens from almost each patient intraoperatively. In this study, the turbinoplasty was performed by creating a mucosal flap in the IT while carefully preserving the medial part and the posterior end of the turbinate as seen in Fig. 1. Specimens were collected from the anterior, posterior, medial, and lateral sites of either IT, placed in containers, and sent to the histopathology laboratory for analysis. All specimens were obtained from the histopathology laboratory after being immediately fixed in 10% buffered formalin. After adequate fixation, the tissue was processed in an auto-processor and embedded in paraffin. A minimum of three levels of slides stained with hematoxylin and eosin were assessed by the same pathologist.

**Figure 2:**
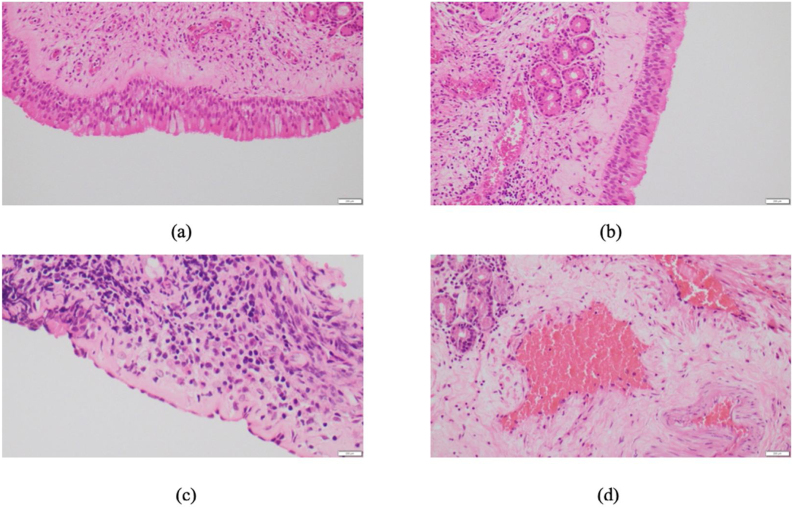
Microscopic images showing the following: (a) depict hyperplastic respiratory epithelium with increased goblet cells, overlaying an inflamed stroma (H&E, 20×), (b) preserved cilia of the respiratory mucosa (H&E, 20×), (c) denude partially denuded mucosa, overlaying an inflamed stroma with eosinophils (H&E, 40×), and (d) an edematous stroma harboring ectatic vessels (H&E, 20×).

To obtain histopathological data, the following parameters were studied and analyzed: nature of the epithelium; basement membrane thickness; ciliary cell status; vessel wall thickness; presence of goblet cells; degrees of edema and fibrosis; presence of acute or chronic inflammation and relevant degree of that inflammation; and presence of eosinophils as shown in Fig. 2.

### Other variables

Demographic variables included sex, age, allergic rhinitis, cigarette smoking, alcohol consumption history, surgical history, and comorbidities. All included patients were evaluated to determine if they had any of the following allergy symptoms: sneezing; rhinorrhea; itchy nose, palate, or throat; and itchy, watery, or red eyes with nasal obstruction. Additionally, the total serum IgE was measured using a 1470 WIZARD gamma counter (PerkinElmer, Turku, Finland) and Immuno CAP100 (Phadia, Uppsala, Sweden). The total serum IgE detected ranged from ≥2.00 to ≤5000.00 kU/L. A diagnosis of allergic rhinitis was made when the total serum IgE level was more than 100 kU/L. Therefore, allergic rhinitis was determined based on the presence of a history of allergies and total serum IgE level more than 100 kU/L.

Patients were categorized as cigarette smokers (more than five packs during their lifetime) or non-smokers (never smoked or had smoked fewer than five packs during their lifetime) based on their lifetime smoking history. Their alcohol consumption history was assessed using the Alcohol Use Disorders Identification Test: a score more than 8 indicated a history of alcohol consumption for men; however, a score ≥4 indicated a history of alcohol consumption for women. The medical history was obtained from all patients, including those with chronic diseases such as diabetes or hypertension (Table 1).

**Table 1:** Comparison of clinic-surgical characteristics between the hypertrophied and non-hypertrophied inferior turbinate.

	Hypertrophied inferior turbinate (n = 30)	Non-hypertrophied inferior turbinate (control group) (n = 4)	*p-*Value
Mean age (mean ± SD)	31.61 ± 12.4	29.75 ± 14.2	0.780
Gender			
Male	20 (66.67%)	4 (100.0%)	0.276
Female	14 (46.67%)	0 (0.00%)	
Allergic rhinitis	7 (23.33%)	0 (0.00%)	1.000
Smoking	10 (33.33%)	2 (50.0%)	0.577
Alcohol	0 (0.00%)	0 (0.00%)	‒
Surgical history	0 (0.00%)	0 (0.00%)	‒
Comorbidities	3 (10%)	0 (0.00%)	1.000

### Statistical analysis

Data were entered, processed, and analyzed using Statistical Package for Social Sciences (SPSS) Statistics for Windows/Macintosh (version 22.0, SPSS Inc., Chicago, IL, USA). Descriptive data are presented as the mean ± Standard Deviation (SD) for continuous variables. Categorical variables are presented as frequencies and percentages. Bivariate analyses were computed through *t*-tests and Fisher’s exact tests where appropriate. Differences were considered statistically significant when *p* < 0.05.

## Results

Our cohort comprised 34 patients, in which 30 patients were in the IT hypertrophy group and 4 patients were in the non-IT hypertrophy group. Of these patients, 24 (70.6%) were men. Patient ages ranged from 18 to 65 years with a mean age of 31.4 ± 12.38 years in the case group and 29.75 ± 14.2 years in the control group. Table 1 shows the demographic data of all patients included in our study which were divided to create the IT hypertrophy group and non-IT hypertrophy group (control group).

### Comparisons of the IT histopathology at four sites

#### Epithelium

As seen in Table 2, we compared the different specimen sites to evaluate the appearance of the epithelium and found no significant differences between the IT hypertrophy group and non-IT hypertrophy group in different specimens (*p* = 0.1). However, normal epithelium was observed most often in specimens obtained from the posterior site (46.4%), whereas focal denudation was observed most often in specimens obtained from the anterior site (53.3%).

**Table 2:** Specimen characteristics comparison between the hypertrophied and non-hypertrophied inferior turbinate.

Characteristics	Findings	Anterior		Posterior		Medial		Lateral	
		Case	Control	Case	Control	Case	Control	Case	Control
Epithelium	Focally denuded	16 (53.3%)	3 (75.0%)	13 (46.4%)	3 (75.0%)	12 (44.4%)	3 (75.0%)	15 (50.0%)	2 (50.0%)
	Completely denuded	4 (13.3%)	0 (0.0%)	1 (3.6%)	0 (0.0%)	8 (29.6%)	0 (0.0%)	3 (10.0%)	2 (50.0%)
	Normal	9 (30.0%)	1 (25.0%)	13 (46.4%)	1 (25.0%)	7 (25.9%)	1 (25.0%)	10 (33.3%)	0 (0.0%)
	Hyperplasia	1 (3.3%)	0 (0.0%)	1 (3.6%)	0 (0.0%)	0 (0.0%)	0 (0.0%)	2 (6.7%)	0 (0.0%)
	*p-*Value	0.102							
Goblet cells	Less	23 (76.7%)	2 (50.0%)	14 (50.0%)	2 (50.0%)	21 (77.8%)	3 (75.0%)	17 (56.7%)	3 (75.0%)
	More	1 (3.3%)	0 (0.0%)	3 (10.7%)	1 (25.0%)	1 (3.7%)	0 (0.0%)	4 (13.3%)	0 (0.0%)
	Normal	6 (20.0%)	2 (50.0%)	11 (39.3%)	1 (25.0%)	5 (18.5%)	1 (25.0%)	9 (30.0%)	1 (25.0%)
	*p-*Value	0.653							
Cilia	Seen	16 (53.3%	2 (50.0%)	21 (75.0%)	2 (50.0%)	12 (44.4%)	3 (75.0%)	21 (70.0%)	3 (75.0%)
	Not Seen	14 (46.7%)	2 (50.0%)	7 (25.0%)	2 (50.0%)	15 (55.6%)	1 (25.0%)	9 (30.0%)	1 (25.0%)
	*p-*Value	0.178							
Basement membrane	Normal	8 (26.7%)	2 (50.0%)	3 (10.7%)	3 (75.0%)	10 (37.0%)	2 (50.0%)	3 (10.0%)	2 (50.0%)
	Thickened	22 (73.3%)	2 (50.0%)	25 (89.3%)	1 (25.0%)	17 (63.0%)	2 (50.0%)	27 (90.0%)	2 (50.0%)
	*p-*Value	0.005*							
Vessels	Normal	14 (46.7%)	3 (75.0%)	11 (29.3%)	3 (75.0%)	14 (51.9%)	3 (75.0%)	13 (43.3%)	3 (75.0%)
	Thickened	16 (53.3%)	1 (25.0%)	17 (60.7%)	1 (25.0%)	13 (48.1%)	1 (25.0%)	17 (56.7%)	1 (25.0%)
	*p-*Value	0.03*							
Edema	Mean	0.6667	0.5	1.1724	1	0.8571	0.5	1.2333	0.75
	*p-*Value	1.2333							
Fibrosis	Mean	1.1667	0.5000	0.9655	0.0000	1.0714	0.5000	0.7667	0.5000
	*p-*Value	0.001*							
Chronic inflammation	Mean	1.2667	0.7500	1.3793	1.5000	1.0714	1.0000	1.2333	1.2500
	*p-*Value	0.456							
Acute inflammation	Mean	0.3000	0.5000	0.3103	0.5000	0.1786	0.5000	0.2333	0.5000
	*p-*Value	0.093							
Eosinophils	Mean	0.0333	0.0000	0.0690	0.0000	0.0714	0.2500	0.0333	0.0000
	*p-*Value	0.852							

* p < 0.05.

#### Basement membrane

IT hypertrophy group’s Specimens with the thickest basement membrane were from the lateral site (90.0%), and the next thickest basement membranes were found in specimens from the posterior site (89.3%). Most anterior site specimens in the IT hypertrophy group had a thickened basement membrane (73.3%). Specimens in the IT hypertrophy group with the least thickness (63.0%) were from the medial site. When comparing the basement membrane thickness between the IT hypertrophy group and non-IT hypertrophy group, the difference was statistically significant (*p* = 0.01) as shown in Table 2.

#### Vessel wall thickness

Thickened blood vessel walls were observed mostly in specimens obtained from the posterior site in the IT hypertrophy group (60.7%) followed by the lateral site (56.7%). On the other hand, they were least often observed in medial site specimens of the IT hypertrophy group (48.1%) as shown in Table 2. When comparing the vessel wall thickness between the IT hypertrophy group and non-IT hypertrophy group, the difference was statistically significant (*p* = 0.03).

#### Ciliary cells status

In the subgroup analysis of the IT hypertrophied group, cilia were observed mainly in posterior site specimens (75.0%). The medial site specimens had the least preserved cilia (44.4%). However, there was no significant difference between the IT hypertrophy group and non-IT hypertrophy group in the presence of cilia (*p* = 0.17) as shown in Table 3.

**Table 3:** Frequency and percent of specimens that cilia were seen in the IT hypertrophied group.

Specimens	Seen	Not seen	*p-*Value
Anterior	16 (53.3%)	14 (46.7%)	0.171
Posterior	21 (75.0%)	7 (25.0%)	
Medial	12 (44.4%)	15 (55.6%)	
Lateral	21 (70.0%)	9 (30.0%)	

### Presence of goblet cells

Comparisons of goblet cell findings among the different areas of the IT hypertrophied group revealed no significant difference among the samples (*p* = 0.11). However, medial site specimens most often had fewer than normal goblet cells, while the lateral site specimens had the highest number of cells, as shown in Table 4.

**Table 4:** Counts of goblets cells in different specimens in the IT hypertrophied group.

Specimen	Less	More	Normal	*p-*Value
Anterior	23 (76.7%)	1 (3.3%)	6 (20.0%)	0.108
Posterior	14 (50.0%)	3 (10.7%)	11 (39.3%)	
Medial	21 (77.8%)	1 (3.7%)	5 (18.5%)	
Lateral	17 (56.7%)	4 (13.3%)	9 (30.0%)	

### Degrees of edema and fibrosis

**Figure 3:**
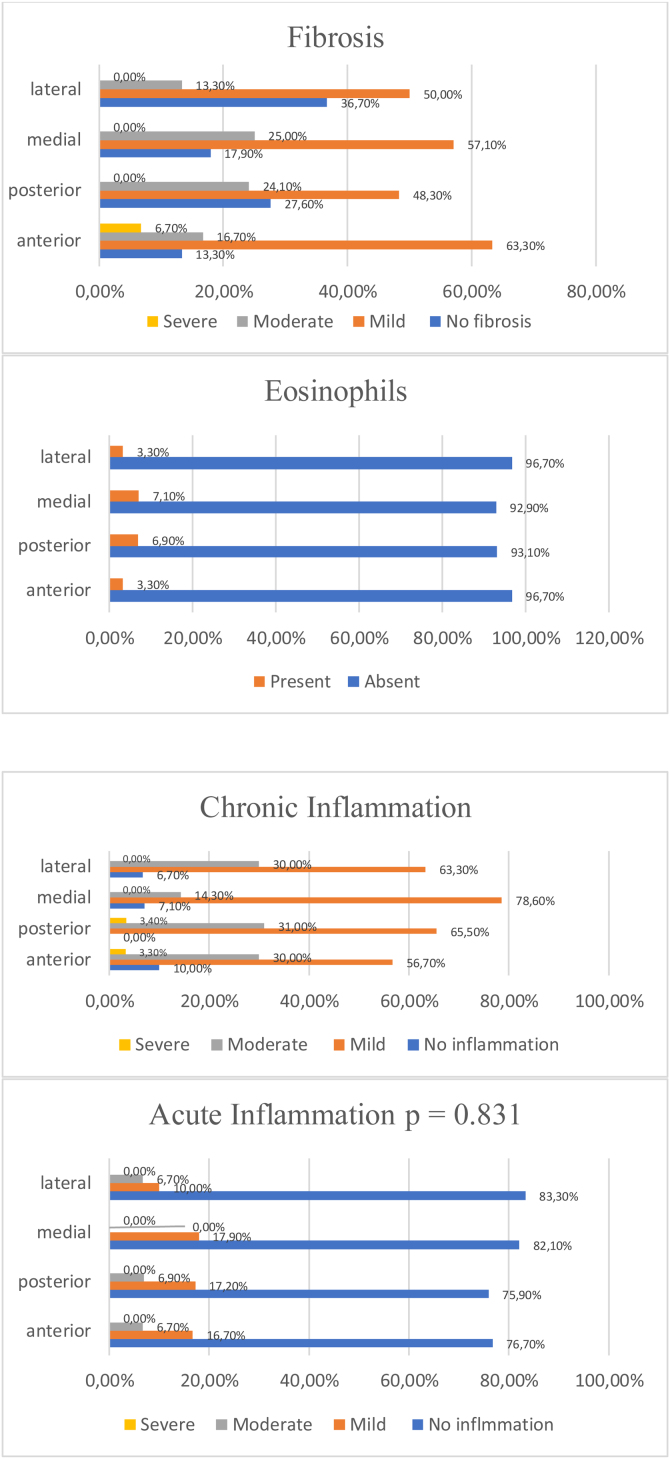
Frequency and presence of fibrosis, eosinophils, chronic inflammation, and acute inflammation in the IT hypertrophied group.

In the subgroup analysis of the IT hypertrophied group regarding the degree of edema, anterior and posterior site specimens exhibited no edema (46.7% and 14.3%, respectively). Furthermore, 48.1% of the medial site specimens exhibited mild edema, 33.3% exhibited no edema, and 18.5% exhibited signs of moderate edema. Finally, severe edema was observed only in one specimen from the lateral site, with mild edema in 53.3%, moderate edema in 30.0%, and no edema in 13.3% of those specimens. However, there was no significant difference among the groups (*p* = 0.08) as shown in Table 5. A moderate degree of fibrosis was found mainly in the anterior site specimens of the IT hypertrophied group (63.3%). The anterior site specimens were the only specimens that showed a severe degree of fibrosis (6.7%). However, no significant difference was observed (*p* = 0.20) as shown in Fig. 3.

**Table 5:** Degree of edema among different specimens in the IT hypertrophied group.

Specimen	No edema	Mild edema	Moderate edema	Severe edema	*p-*Value
Anterior	14 (46.7%)	12 (40.0%)	4 (13.3%)	0 (0.0%)	0.081
Posterior	4 (14.3%)	15 (53.6%)	9 (32.1%)	0 (0.0%)	
Medial	9 (33.3%)	13 (48.1%)	5 (18.5%)	0 (0.0%)	
Lateral	4 (13.3%)	16 (53.3%)	9 (30.0%)	1 (3.3%)

### Presence of acute or chronic inflammation and eosinophils

Mild chronic inflammation was observed more often than moderate and severe chronic inflammation in the IT hypertrophied group. Furthermore, mild chronic inflammation was found mainly in medial site specimens of the IT hypertrophied group (78.6%). Severe chronic inflammation was found only in anterior and posterior site specimens (3.3% and 3.4%, respectively). No significant difference was observed between specimens (*p* = 0.56). In contrast to chronic inflammation, acute inflammation was absent from almost all specimens. There was no significant difference among the groups (*p* = 0.83) as shown in Fig. 2. The site with the most eosinophils was the medial site, followed by the posterior site as shown in Fig. 2.

### Comparisons of the IT hypertrophy and non-IT hypertrophy groups

Specimens from the control group (n = 4) revealed no differences except for the basement membrane thickness; specimens taken from the posterior site, unlike those from the IT hypertrophy group, had basement membranes with normal thickness (Table 6). A comparison between the IT hypertrophy group samples and those of the control group showed no significant differences in epithelium, goblet cells, edema, chronic inflammation, acute inflammation, and eosinophils. However, the IT hypertrophy group samples had a significantly higher prevalence of thickened basement membranes and thickened vessels than those of the control group (*p* = 0.005 and *p* = 0.03, respectively).

**Table 6:** Characteristics of specimens taken from the control group.

		Anterior	Posterior	Medial	Lateral	*p-*Value
Epithelium	Focally denuded	3 (75.0%)	3 (75.0%)	3 (75.0%)	2 (50.0%)	0.481
	Completely denuded	0 (0.0%)	0 (0.0%)	0 (0.0%)	2 (50.0%)	
	Normal	1 (25.0%)	1 (25.0%)	1 (25.0%)	0 (0.0%)	
Goblet cells	Less	2 (50.0%)	2 (50.0%)	3 (75.0%)	3 (75.0%)	0.559
	More	0 (0.0%)	1 (25.0%)	0 (0.0%)	0 (0.0%)	
	Normal	2 (50.0%)	1 (25.0%)	1 (25.0%)	1 (25.0%)	
Cilia	Seen	2 (50.0%)	2 (50.0%)	1 (25.0%)	1 (25.0%)	0.171
	Not seen	2 (50.0%)	2 (50.0%)	3 (75.0%)	3 (75.0%)	
Basement membrane	Normal	2 (50.0%)	3 (75.0%)	2 (50.0%)	2 (50.0%)	0.01*
	Thickened	2 (50.0%)	1 (25.0%)	2 (50.0%)	2 (50.0%)	
Vessels	Normal	3 (75.0%)	3 (75.0%)	3 (75.0%)	3 (75.0%)	0.623
	Thickened	1 (25.0%)	1 (25.0%)	1 (25.0%)	1 (25.0%)	
Edema	No edema	2 (50.0%)	1 (25.0%)	2 (50.0%)	1 (25.0%)	0.486
	Mild edema	2 (50.0%)	2 (50.0%)	2 (50.0%)	3 (75.0%)	
	Moderate edema	0 (0.0%)	1 (25.0%)	0 (0.0%)	0 (0.0%)	
	Severe edema	0 (0.0%)	0 (0.0%)	0 (0.0%)	0 (0.0%)	
Fibrosis	No fibrosis	2 (50.0%)	4 (100.0%)	2 (50.0%)	2 (50.0%)	0.140
	Mild fibrosis	2 (50.0%)	0 (0.0%)	2 (50.0%)	2 (50.0%)	
	Moderate fibrosis	0 (0.0%)	0 (0.0%)	0 (0.0%)	0 (0.0%)	
	Severe fibrosis	0 (0.0%)	0 (0.0%)	0 (0.0%)	0 (0.0%)	
Chronic inflammation	No inflammation	1 (25.0%)	0 (0.0%)	0 (0.0%)	0 (0.0%)	0.801
	Mild inflammation	3 (75.0%)	2 (50.0%)	4 (100.0%)	3 (75.0%)	
	Moderate inflammation	0 (0.0%)	2 (50.0%)	0 (0.0%)	1 (25.0%)	
	Severe inflammation	0 (0.0%)	0 (0.0%)	0 (0.0%)	0 (0.0%)	
Acute inflammation	No inflammation	2 (50.0%)	2 (50.0%)	2 (50.0%)	2 (50.0%)	0.451
	Mild inflammation	2 (50.0%)	2 (50.0%)	2 (50.0%)	2 (50.0%)	
Eosinophils	Present	4 (100.0%)	4 (100.0%)	3 (75.0%)	4 (100.0%)	0.709
	Absent	0 (0.0%)	0 (0.0%)	1 (25.0%)	0 (0.0%)	

* p < 0.05.

## Discussion

IT hypertrophy is considered the main structural cause of chronic nasal obstruction. Underlying problems such as allergic and non-allergic rhinitis can aggravate the severity of nasal obstruction. Therefore, various surgical techniques have been used to achieve optimal volume reduction of IT hypertrophy.^8^ The primary aim of surgical intervention is to increase the nasal airway patency, thus improving breathing, preserving the IT function, and preventing possible complications. Because surgical procedures are being developed to preserve function, histological analyses of the IT are imperative. During the current study, we compared the histological and morphometric differences between the control and IT hypertrophy groups, thus providing us with a better understanding of the pathophysiological mechanisms of IT enlargement to improve and advance procedures aimed at reducing IT hypertrophy.

Several studies have investigated IT histopathology. Burger et al. studied the histopathological features of the IT and its multiple tissue components (e.g., connective tissue, epithelium, arteries, venous sinusoids, and submucosal glands); however, their study was based on autopsy results and did not aim to modify the surgical technique.^9^ Our study, however, involved histopathological analyses of living patients, and variables of the functional status of the IT were studied and analyzed.

The mucosa of the IT, which is pivotal in hypertrophic changes, comprises the respiratory epithelium and is involved in immunological defense. Therefore, IT has an important role in protecting the nasal cavity against exogenous stimuli, such as allergens, chemical irritants, infectious agents, and temperature changes. During the current study, the highest normal epithelium percentages and cilia counts were found in the posterior end of the IT. Because these elements are vital for the normal function of IT, it is of great importance to the mucociliary function to protect the ciliated epithelium function. Therefore, based on these findings, preserving the posterior part must be considered during surgical modification.^9^

According to Berger et al., the medial mucosal layer was thicker than the bone and lateral mucosa, which was statistically significant.^3^ However, during our study, we found that the lateral part was thickest in the basement membrane and consisted of more goblet cells, whereas the medial part had fewer than normal goblet cells. However, the findings of Mogensen and Tos were corroborated by our study results, which showed that more goblet cells were found in the lateral wall.^6^ Moreover, they reported an inverse ratio of the number of goblet cells and the strength of the air current, and they showed that their number was abundant in regions where airflow is sparse, and vice versa. Therefore, it may be necessary to retain the medial part and discard the lateral part of the IT to obtain better surgical outcomes.^10^

Venous sinusoids have an important role in IT pathology. Schmidt et al. found a greater concentration of venous sinusoids in the inferior mucosal layer than in the medial and lateral mucosal layers. Therefore, the inferior mucosal layer should be considered an appropriate site for turbinate reduction to avoid the effects of excessive congestion of venous sinusoids.^11^ According to our study results, the thickest blood vessel walls were found more often on the posterior and lateral sides and least often on the medial side. This fact may have important functional implications because excision of the posterior and lateral portions is associated with a higher risk of intraoperative bleeding.^12^

It has been reported that the number of inflammatory cells in the mucosa and hyperplasia of blood vessels are positively associated with mucosal edema. In contrast, the severity of fibrosis in the mucosa is negatively associated with the presence of mucosal edema. During our study, a moderate degree of fibrosis was found mostly in anterior specimens. Based on these results, it is feasible to address the anterior part of the IT with no significant effect on the function. Moreover, because the head of the IT contributes to the cross-sectional area of the nasal valve, any surgical reduction of the anterior part of the IT would contribute to relieving the nasal obstruction symptoms and provide a good nasal airway.^13^, ^14^

As shown by our results, there were statistically significant differences in the prevalence of thickened basement cells and thickened vessels in the IT hypertrophy group compared to the control group (*p* = 0.005 and *p* = 0.03, respectively). These results are consistent with the findings of basement membrane thickening in patients with allergic rhinitis and chronic rhinosinusitis.^15^, ^16^, ^17^ Moreover, postoperative restoration of the basement membrane to a normal state can be achieved with reduction procedures performed to reduce inflammation, thus supporting the theory of remodeling occurring in the upper airways.^7^, ^18^

Our study was limited by the dynamic features of the IT that occur during the nasal cycle and could change the IT size. To overcome this limitation, we included perennial allergic rhinitis patients with persistent and severe nasal symptoms that did not respond to medical treatment and required surgical intervention to improve nasal airway patency. Another limitation was the possible overlap of infectious and allergic symptoms. Although there was a high possibility of enrolling patients with infectious rhinitis instead of allergic rhinitis which could result in information bias, we overcame this issue by obtaining detailed patient histories and performing confirmative tests such as total serum IgE level measurements. Moreover, patients usually present with long-term symptoms; therefore, the possibility of mucosal thickening was predominantly high.

## Conclusion

In conclusion, the results of this study strongly suggest that adequate knowledge of nasal IT histology is essential for planning the surgical approach because it may strongly affect the outcomes. Therefore, further studies such as randomized trials with follow-ups of the enrolled patients are needed. Moreover, data collected during this study can be utilized in future studies to support the showed outcomes and to elucidate the efficacy of the IT surgical interventions.

## Funding

This research did not receive any specific grant from funding agencies in the public, commercial, or not-for-profit sectors.

## Conflicts of interest

The authors declare no conflicts of interest.
